# The British Journals: Pathology, Practical Medicine, and Therapeutics

**Published:** 1840-04

**Authors:** 


### PATHOLOGY, PRACTICAL MEDICINE, AND THERAPEUTICS.

*Cases of Mortification of the Male Genitals, giving rise (as is supposed) to fatal Fever by Infection.* By ROBERT PALEY, M.D.

[THE following narrative is very interesting and well worthy the attention of pathologists. The evidence of the infection is not unquestionable, but we are disposed to adopt Dr. Paley's views.]

The first case was that of a stout man, aged thirty, by profession a teacher of music, residing in a village near Halifax, and a married man of very regular habits; the surgeon who was in attendance informed me that he had seen him, for the first time, on the preceding day, and that the patient informed him that it was only on the previous day that he had first felt some uneasiness in the scrotum; that it very soon became enlarged and inflamed, in which state it was when the surgeon first saw it; he immediately applied a dozen leeches, and afterwards ordered fomentations and purgatives. On the following day I was consulted, being the third day from the commencement, when the whole of the scrotum and the penis had assumed a dark red colour, with here and there a black gangrenous spot; the tongue was dark coloured as in typhus, and the pulse indicated debility. We agreed to give our patient decoction of bark with porter, and to apply an ale poultice, with lint dipped in melted unguentum resinæ flavæ. This plan was continued, with the occasional addition of sp. terebinthinæ to the ointment, until the whole of the scrotum, prepuce, and a considerable part of the penis, sloughed off. The patient's strength was supported, and in the course of a few weeks he recovered so far as to live for seventeen or eighteen years, when he died, I believe, of phthisis. His death took place some years after I had left that neighbourhood.

The second case was that of a farmer, about thirty-five years of age, residing a few miles from Ripon, who thought that he had caught cold, which had brought on some itching of the penis and scrotum, both of which, in the course of two days, became very much inflamed and enlarged; on which account he sent for a surgeon, who took blood from the arm, applied fomentations, and gave him purgative medicines. On the following day, to his great surprise and dismay, a gangrenous spot appeared on the scrotum. The blood taken exhibited no indication of inflammation. Before he left the house I saw the patient with him, and told him what, in my opinion, would be the result, which he could scarcely credit: I then gave him an account of the former case, and, as far as lay in my power, put him on his guard. In this case the tongue exhibited the same appearances as in the former. We gave decoction of bark in the porter, and applied an ale poultice, with dressings, similar to those employed in the former case. The inflammation extended along the abdomen as high as the umbilicus, above which there was an eruption resembling the ecthyma cachecticum of Willan. Suppuration took place above the pubes, the whole of scrotum and prepuce sloughed off, and the patient is convalescent.

During my attendance in the first case, one morning, whilst the surgeon was dressing the patient, the scrotum and penis being in a gangrenous state, a messenger came to request him to go to a woman in labour, who resided about half a mile from our patient, and he obeyed the summons without loss of time. Four or five days after this, on meeting again, he said, "you will recollect that I was sent for to a woman in labour on such day." I replied yes, what of that? "She is dead; everything seemed to be going on well until yesterday, when she was seized with violent pain in the region of the uterus, and she died before I had time to do anything to relieve her." In the course of two or three days, on meeting again, he said, "It is very odd, Dr. Paley, I have lost another patient in the same unaccountable way as before;" and the next morning, at our meeting, he stated that he had another patient about two miles off seized in the same manner, whom he requested me to visit along with him. After seeing his patient, I told him that she was labouring under puerperal fever, and before we left the house he was sent for to visit another woman whom he had attended in labour in the same village. I accompanied him, and found her also the subject of puerperal fever. I believe that he had in all six cases of this disease.

I enquired of nearly all the general practitioners in Halifax and the neighbourhood if they had any cases of puerperal fever, but not one could I hear of; indeed, most of the medical men owned that they had never seen a case of it in the whole of their practice. About the same time I was requested to visit a married lady, aged fifty-four, who resided in the same village as the teacher of music, betwixt whom, however, there had not been any intercourse, but she had visited repeatedly the first woman who had died so unexpectedly. I found that she had been seized, on the day previous to my seeing her, with violent pain of the bowels, which had continued to increase in spite of the means employed. When I arrived she was *in articulo mortis*, and expired before I left the room, which was twenty-six hours after the time she had first felt any sort of uneasiness. In the present day this case would very probably have been considered as Asiatic cholera; and it is to be regretted that we were not permitted to make a post-mortem examination.

There is not the slightest doubt on my mind that the surgeon who was in attendance was the means of communicating something (call it what you please) from the patient labouring under the disease of the scrotum to the lying-in women, which in them produced puerperal fever; and with regard to the lady last mentioned, the first case of disease after parturition, which in all probability was that of puerperal fever, produced in her a species of enteritis, which in its progress bore a considerable resemblance to the disease of the music-master.

I pointed out these circumstances to the surgeon, and at the same time advised him to go from home two or three weeks, and to have his clothes washed and fumigated; he did so, and the plague (for such it seemed) ceased. These circumstances I also mentioned to the surgeon in attendance on the case which has recently occurred in this neighbourhood. I advised him to wash his hands well previous to leaving the house of his patient, and not to attend any woman in labour or after her confinement, without first changing his dress. Notwithstanding this precaution, which I believe he rigidly observed, I received a note from him a few days ago, stating that he had some unfortunate cases of puerperal fever.

*London Medical Gazette. Dec.* 6, 1839.

---

*Cases and Observations on Anuria Apyretica.* By P. CAMPBELL, A.M., Surgeon.

[UNDER this title the author details, in a very lucid manner, the particulars of three fatal cases of the disease better known under the Cullenian name of *ischuria renalis.* As we have not room to copy them, we refer to them chiefly for the sake of young practitioners, whom we have often had occasion to see misled by the insidious and seemingly mild invasion of this deadly disease. Mr. Campbell's cases terminated in the usual manner, within a short period after the formal invasion of the malady. We quote the two concluding paragraphs of his well-written communication:]

A minute investigation, had it been permitted, might have discovered different morbid lesions to have accounted for the invasion of the disease in each of these three individuals, but the fatal termination was the same in all of them; profound coma, laborious, labial, occasional irregular respiration, with viscid frothy saliva, but without any symptom of palsy or dropsical effusion. Whether, therefore, we trace the origin of the disease to the solids or fluids, whether, from a paralytic state of the kidneys, their secreting functions become suspended, and the effete matter retained in the system; or whether, on the urine being secreted, but obstructed in its course to the bladder, and consequently reabsorbed into the circulation, the impure blood seems to act as a morbid poison on the brain, sufficiently powerful to occasion death, independent of effusion.

It unfortunately happens that, in cases of anuria, as in many other instances, the approach is so very insidious and masked, that the disease is not even suspected till it be fully formed, and the proper period for active treatment past. Prompt and free bleeding, both generally and locally; the warm bath with friction; stimulating external applications; demulcent, rather than irritating, diuretics, with purgatives calculated to produce watery digestions, seem to be the most likely means to combat this obscure but fatal malady.

*Lancet. Dec.* 14, 1839.

---

*On the Treatment of Croup by large doses of Tartar Emetic.*
By CHARLES WILSON, M.D., Kelso.

I HAVE now treated in all twelve cases of croup in children with high doses of tartrate of ammonia on the contra-stimulant plan, and of these two only have died. In one of the fatal cases there had been distinct and severe croupy symptoms for two days before I had any opportunity of exhibiting the medicine, yet its use was followed by a striking relief of these symptoms. Within the first seven days of treatment, the patient, a boy of four and a half years of age, took thirty-six grains of the tartrate, in doses which averaged half a grain, given at first every hour, and afterwards every two hours. The cough and respiration improved from the beginning, and at the close of the fifth day of treatment resembled what is heard in ordinary catarrh. The voice improved on the sixth day, and at the close of the eighth day of treatment it was heard distinctly, though previously little more than a feeble whisper. The medicine frequently caused vomiting, but no catharsis; the bowels, on the contrary, requiring to be regulated by enemata. On the eleventh day there was a relapse, and on the twelfth day the cough was again croupy and the inspirations sonorous. I hesitated, and I believe improperly, in again employing the antimony; and the patient died on the fifteenth day after I had first seen him, and the seventeenth from the commencement of the disease. In the other case the patient died on the fourth day of the treatment, the antimony, however, having never been properly administered, nor my directions in other respects implicitly followed. With respect to the successful cases, I may observe, that I certainly met, for the most part, with little tolerance of the remedy in as far as the stomach was concerned, vomiting having been freely produced in nearly all; but there was no troublesome catharsis, and, even in one instance where there was a tendency to diarrhœa previously to the exhibition of the antimony, this ceased on the second day of the treatment, and it became necessary to have recourse to mild laxatives. Indeed, not a single case occurred, during some part of the treatment of which it was not requisite to exhibit castor oil, infus. sennæ, or some other remedy to act upon the bowels. The treatment was generally commenced with the application of leeches to the larynx, which were followed by warm poultices, frequently renewed; and, simultaneously with the leeches, the tartar-emetic was begun in doses of one fourth or one third of a grain, generally at first every hour till a decided impression was made, and afterwards every two hours till the patient was considered in safety; care being taken that its use was not intermitted at too early a period for the certainty of the cure. The remedy was usually exhibited in a mixture with a little mucilage, prepared with warm water to ensure its solution; and occasionally, with the older children, half a minim or a minim of T. Opii was joined to each dose, which seemed to have a marked effect in ensuring tolerance of the medicine, without diminishing its sanatory effects. The largest quantity given in any of the successful cases was sixteen grains, and in this case the tolerance was complete after the first two doses. In one of the cases the antimony, at a certain period, was combined with calomel, of which upwards of a drachm was exhibited. A blister was generally applied to the top of the sternum towards the close of the treatment, in order to obviate the risk of a relapse.

I am well aware that the tart. of antimony has been employed by numberless practitioners in croup, and that Cheyne among the rest has prescribed it in considerable and frequently repeated doses; but I am not aware that any one has exhibited it in precisely the same way or with the same aims as myself, or that they have sought from it anything beyond its ordinary well-ascertained action as an emetic. I have thus had no guide to direct me in the use of the remedy, an advantage which I probably might have possessed had my acquaintance with medical literature been more extensive; and, of course, I do not pretend that the limited experience which I have had of it has established its value beyond dispute. Having formed my diagnosis, however, with what care I could, and having treated my cases at periods when I heard of others attended with fatal consequences occurring in the vicinity, and my success in the treatment having been far greater than any I had hitherto attained with other methods, I considered myself justified in laying these details before you, and in recommending the remedy to your attention. *Border Med. Society.* 1839.

---

### *Contributions to the Pathology of Children. Tubercles of the Brain.* By P. Hennis Green, m.b.

[We have only room for the *Remarks* appended to these five valuable cases. These remarks merit the best attention of pathologists and practitioners.]

The first circumstance which strikes us is, that tuberculous deposit, even to a very great extent, may exist in any part of the cerebral substance without producing any disturbance of the economy during life. This is a very remarkable fact. If the brain be the organ of the mind, and if (as it seems to be highly probable) different parts of the brain minister to different mental faculties, is it not strange that a portion of the cerebral substance, as large as a closed fist, may be totally disorganized without any influence being exercised on the intellectual faculties or the other functions of the brain? But without speculating let us content ourselves with noticing the fact. In about one sixth of the cases of cerebral tubercles which I have seen there was no symptom whatever of their existence during life. In several others the symptoms were highly equivocal, and might have been attributed to any other cause. Thus, in the case of Mettaver, the only premonitory sign was habitual headach; after this had lasted for a certain time, symptoms very closely resembling those of acute hydrocephalus set in, and terminated fatally. In the other cases, however, the premonitory symptoms were much better marked, and consisted in some remarkable lesion of the motory power. It is here, in fact, that we must look for the essential symptoms of cerebral tubercle: and I believe that whenever long-standing lesions of this function exist in scrofulous subjects, they will generally be found to depend on tubercle of the nervous centres. The history of the case, if accurately noted, will often throw much light on its nature; the parents or relatives of the young patient will be found to have suffered from scrofulous affections, he himself, perhaps, bears external and visible signs of the malady; if the chest be carefully auscultated the presence of tubercles may be detected in that cavity, or we may, perhaps, find that some of the mesenteric ganglia are enlarged. All these circumstances will aid us in the formation of a diagnosis, which, it must be confessed, is extremely difficult. The nature of the malady must be determined mainly after the lesions of motility; yet how various, how uncertain are these; how difficult to connect them together, or refer them to any particular part of the organ which we suppose to be injured.

In our first case the child had some indeterminate cerebral attack at the age of seven, which was followed by amaurosis for nine days. Now, amaurosis is one of the occasional symptoms of cerebral tubercle; but how difficult was it to connect the symptom and its cause in the case of Humanery, to which I now refer. During two years the muscles of the face were occasionally convulsed in a peculiar manner, without any other symptom, and the child died suddenly, with symptoms which resembled those of hydrocephalus in its clot. Here I would beg the reader's attention to a point which seems to me of the very highest importance in the history of cerebral diseases. A great portion of the obscurity and seeming irregularity of cerebral diseases in children depends on our not having observed them accurately; and I am convinced that most of the cases which have been described as cases of "irregular hydrocephalus," of "cerebral inflammation, with abnormal symptoms," were simply nothing more than examples of cerebral tubercle; the symptoms, progress, and termination are exactly the same in both cases.

In the second case, the symptoms produced by the tubercles in their chronic stage, were a peculiar nervous tremor of one side of the body, and then true epilepsy; in the third case, convulsions, with a peculiar rotatory movement of the head; while, in the fifth case, the only premonitory symptom was habitual headach. I had, therefore, some reason to say, that the symptoms of cerebral tubercle were very various and uncertain. Nevertheless, I feel hope that when I shall have placed before my medical brethren a series of forty cases, some advance will have been made towards a better understanding of this difficult question.

*Lancet. Jan.* 18, 1840.

